# Augmented Endoscopic Images Overlaying Shape Changes in Bone Cutting Procedures

**DOI:** 10.1371/journal.pone.0161815

**Published:** 2016-09-01

**Authors:** Megumi Nakao, Shota Endo, Shinichi Nakao, Munehito Yoshida, Tetsuya Matsuda

**Affiliations:** 1 Graduate School of Informatics, Kyoto University, Yoshida Honmachi, Sakyo, Kyoto, Japan; 2 Department of Orthopedic Surgery, Wakayama Medical University, Wakayama, Japan; University of Cambridge, UNITED KINGDOM

## Abstract

In microendoscopic discectomy for spinal disorders, bone cutting procedures are performed in tight spaces while observing a small portion of the target structures. Although optical tracking systems are able to measure the tip of the surgical tool during surgery, the poor shape information available during surgery makes accurate cutting difficult, even if preoperative computed tomography and magnetic resonance images are used for reference. Shape estimation and visualization of the target structures are essential for accurate cutting. However, time-varying shape changes during cutting procedures are still challenging issues for intraoperative navigation. This paper introduces a concept of endoscopic image augmentation that overlays shape changes to support bone cutting procedures. This framework handles the history of the location of the measured drill tip as a volume label and visualizes the remains to be cut overlaid on the endoscopic image in real time. A cutting experiment was performed with volunteers, and the feasibility of this concept was examined using a clinical navigation system. The efficacy of the cutting aid was evaluated with respect to the shape similarity, total moved distance of a cutting tool, and required cutting time. The results of the experiments showed that cutting performance was significantly improved by the proposed framework.

## Introduction

**Fig 1:**
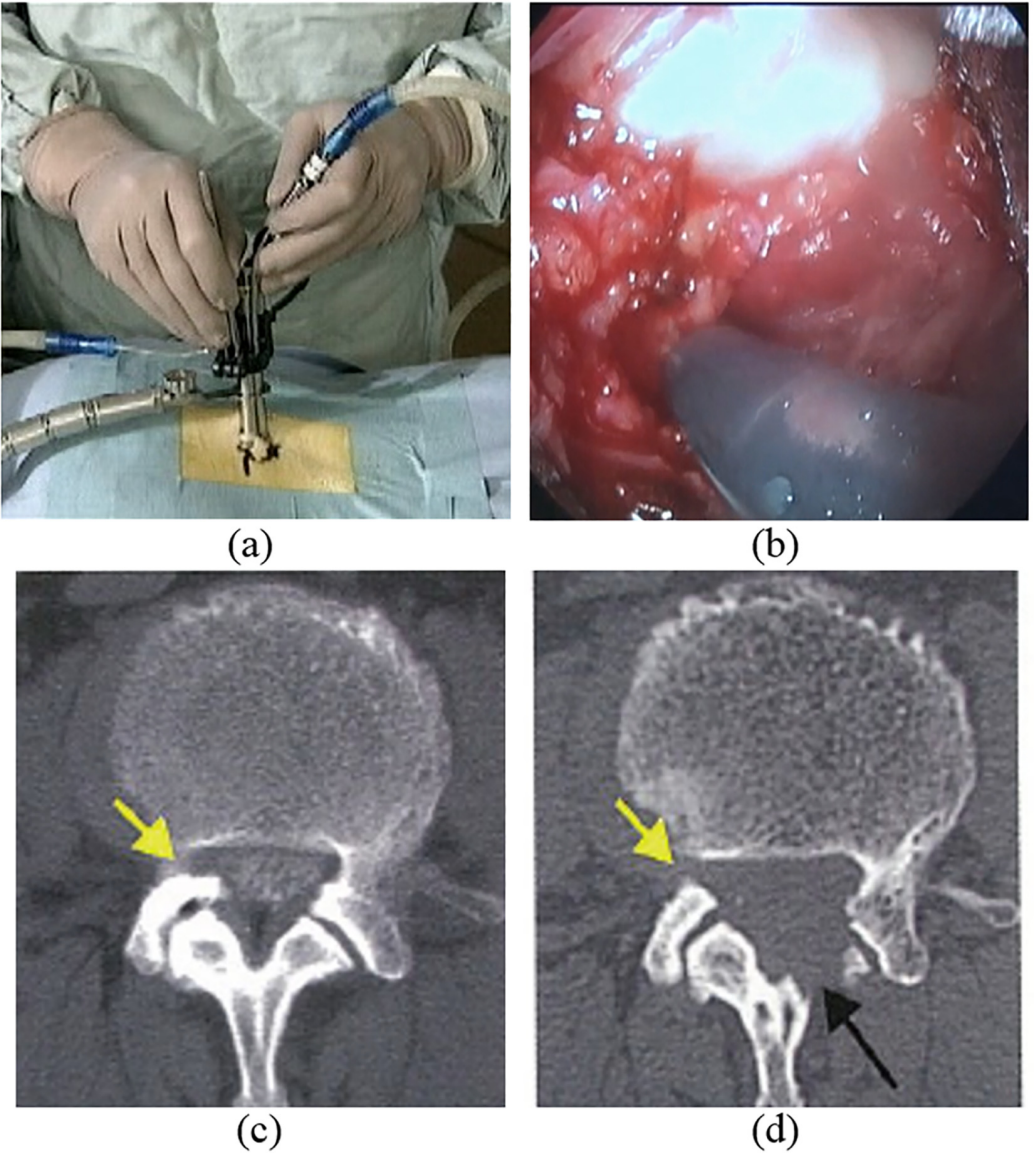
Microendoscopic discectomy for spinal disorders. (a) Tool operation during surgery. (b) Endoscopic view with a 16-mm diameter. (c) Preoperative and (d) postoperative CT images. The yellow and black arrows show the cutting results.

The number of patients with spinal disorders is expected to increase as the population age distribution changes. The spine deforms with age, causing a condition known as lumbar canal stenosis. This condition compresses the nerves located within the lumbar canal and can lead to walking disorders and numbness of the feet. Surgical procedures that incise large areas of skin and implant support devices were traditionally performed to address such issues. Microendoscopic discectomy (MED) [1–3] has recently been performed through small skin incisions and partial cutting of spinal bone structures. Further widespread adoption of MED is expected because this surgical procedure neither requires a lengthy rehabilitation period nor induces an intraoperative burden to the patient. However, MED must be performed with surgical drills in tight spaces while the surgeon observes only a small portion of the target area (See Fig 1). Because two-dimensional (2D) endoscopic images provide visual cues with poor depth information, anatomical knowledge and surgical experience associated with a variety of clinical cases are essential factors for successful procedures. A high degree of technical skill with respect to tool operation and spatial perception are required to treat the target region safely and accurately [4]. Given the high degree of difficulty associated with performing MED procedures, intraoperative support systems are gaining traction as a means for better preservation of the patient’s intervertebral joints and performance of safer surgery.

In the last few decades, intraoperative navigation systems using the patient’s computed tomography (CT) and magnetic resonance images have been developed to support microendoscopic surgery [5,6]. The current location of the surgical tool tip is measured by an optical tracking system and viewed using 2D slice images [7,8]. Although accurate measurement of the surgical tool tip is possible with an error of <0.5 mm, intraoperative information available from conventional surgical navigation products has been limited to visualization of the measured tool tip positions. To visualize three-dimensional (3D) anatomical information, volumetrically rendered images or 3D shape models are also utilized for preoperative planning [9,10], intraoperative navigation [11,12], or medical training [13–15]. However, the spinal structures change as the surgery progresses. Careful bone cutting must be performed by skilled surgeons while they estimate the current shape (e.g., partially-cut vertebra) and the target shape to be cut. Although shape estimation of the target structures is essential for accurate cutting, this estimation depends heavily on spatial perception and the experience of surgeons. The poor shape information available during surgery makes accurate cutting difficult, even when preoperative CT and magnetic resonance images are used for reference. Some studies have reported the use of intraoperative CT [16,17]; however, frequent measurement is required to update the shape model as the bone cutting progresses, which interrupts the procedures and burdens medical staff.

Some studies have focused on applying augmented reality (AR) to intraoperative navigation [18–21]. If the rendered images generated from the preoperative CT volume data are overlaid using AR techniques on the endoscopic images, it becomes easy to understand the corresponding relation to the target structures, making it possible to intuitively and precisely recognize the regions scheduled to be cut. However, AR systems have been designed on the basis of shape changes not occurring during surgery, with the assumption that the target for overlay is a rigid body [20,21]. In recent years, some researchers have sought markerless augmentation [22,23] and visualization of internal tissue (e.g., tumors or blood vessels) for deformable bodies [24]. However, time-varying shape changes or the loss of the tissue during cutting procedures are still challenging issues for intraoperative navigation. Specifically in the case of MED procedures, shape reconstruction from visual information cannot be applied because of the relatively large cutting area with limited visibility. Additionally, conventional semi-transparent representation of virtual images [18,25] may make visual confirmation of real endoscopic images more difficult. Specifically for intraoperative cutting aids, effective information overlay that could suppress the degradation of endoscopic images is desired because the target region for cutting and information overlay is spatially identical. The main focus of this study, therefore, was to resolve these issues in AR navigation for bone cutting procedures.

This paper introduces a concept of endoscopic image augmentation that overlays shape changes in bone cutting procedures for spinal surgery navigation. Our framework estimates the loss of the tissue during cutting based on the history of the drill tip location [26] and generates an augmented endoscopic (AE) image that overlays the remains to be cut. The AE image enables precise cutting by visualizing a differential map between the current state and the target 3D shape spatially registered to the endoscopic image in real time. The feasibility of this concept was examined using a clinical navigation system, and the proposed AE image and the volume-rendered (VR) image of the target shape were compared using an experimental system. The efficacy of the cutting aid was evaluated with respect to the shape similarity, total moved distance of the cutting tool, and required cutting time. The results of the experiments show how cutting performance is improved by the proposed framework.

## Materials and Methods

### Generation of augmented endoscopic image overlaying shape changes

**Fig 2:**
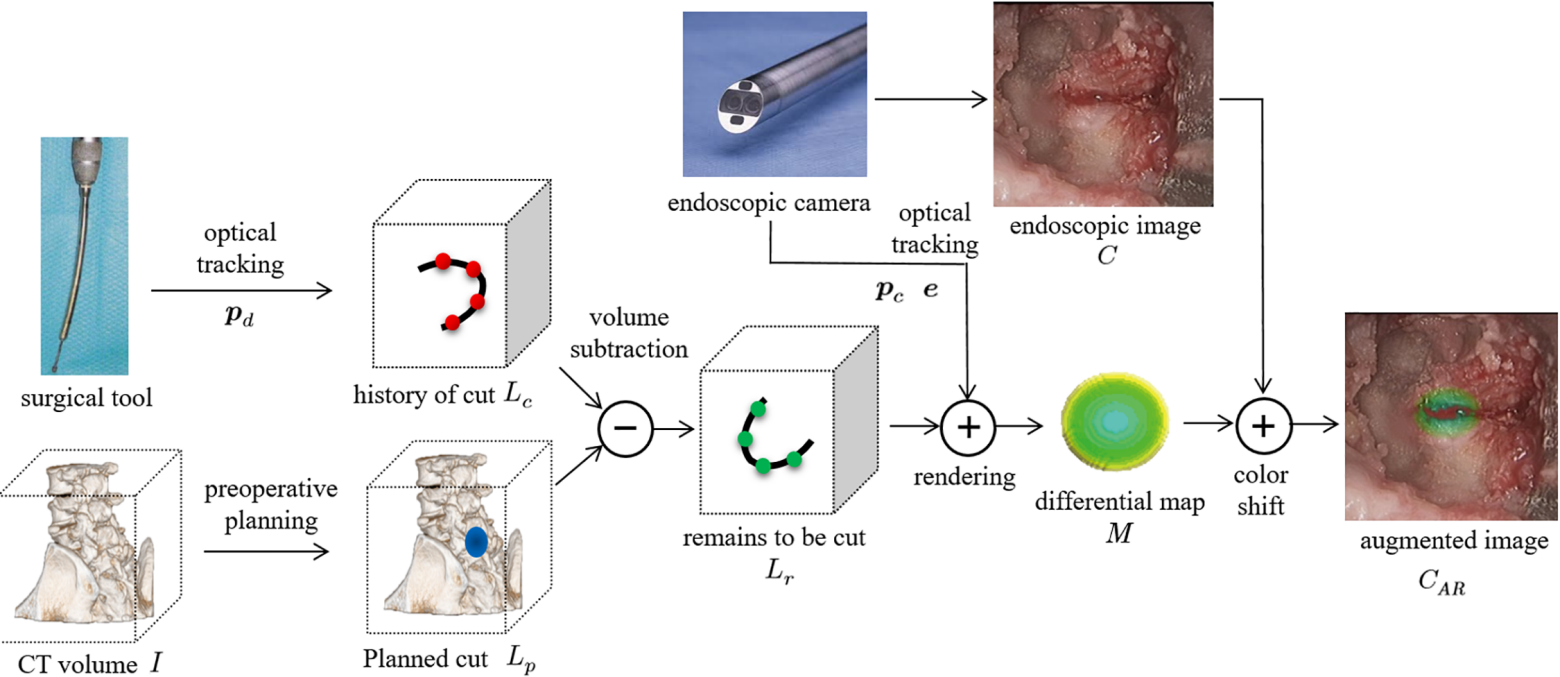
Flowchart of augmented endoscopic image generation. The remains to be cut for the target shape are visualized as augmented images based on the history of the drill tip during cutting.

The proposed AE image overlays the amount of cutting that remains to shape the target on the endoscopic image based on the trajectory of the drill tip during cutting. The processing flow used to generate the AE images is shown in Fig 2. Preoperative planning is first performed to define the 3D region scheduled to be cut *L*_*p*_, which represents the realistic area to be cut during surgery. Interactive virtual cutting [10] is available for planning the cutting area using 2D slice images or VR images of the patient’s CT images *I*. During surgery, 2D endoscopic images *C*(*x*,*y*) with a 16-mm diameter are measured using an endoscopic camera, as shown in Fig 1B. We assume that an optical tracking system such as Polaris (Northern Digital Inc.) is used to measure the orientation ***e*** and location ***p***_*c*_ of the endoscopic camera and the tip of the surgical tool ***p***_*d*_. Rendered images of the spine are then obtained based on the location that was aligned at *C* by rendering the spinal CT volume data *I* while reflecting the camera’s location ***p***_*c*_ and orientation ***e***. It is also possible to visualize the relative position of the surgical tool tip and the spine by displaying ***p***_*d*_ on the rendered images.

The 3D regions that have already been cut are modeled using a volumetric history label *L*_*c*_ with the same size as *I*. The value *l* = 0 is first set for the noncutting voxel in *L*_*c*_. We assume that the voxels where the surgical tool tip has passed within the scheduled region *L*_*p*_ represent the region that has already been cut. The voxel value *l* in *L*_*c*_ is then updated from the relationship between the central location of the arbitrary voxel and the tip position. The remains to be cut are defined using another volume label *L*_*r*_ and updated in real time during surgery. The relationship of these volume labels is simply defined by volume subtraction using Eq (1).

$$L_{r}=L_{p}-L_{c}$$ (1)

Next, the differential map *M*(*x*,*y*) is generated from the cutting label *L*_*r*_, the camera’s location and orientation to represent the difference between the target shape and the current shape. *M* is a 2D projected image generated by volume rendering of *L*_*r*_, and visualizes the remains to be cut in the depth direction registered to the camera image *C*. The AE image *C*_*AR*_(*x*,*y*) is finally generated using *M* by shifting the pixel value of *C* locally. *C*_*AR*_ is the AE image that designates the remains to be cut as a time-varying heat map that is updated as cutting progresses. The proposed framework is summarized in the following four steps:

STEP 1. Obtain the direction for the camera’s line of sight ***e***, the location of the endoscopic camera ***p***_*c*_, and the location of the surgical tool tip ***p***_*d*_ using the optical tracking system. Obtain the camera images *C* via an endoscope.
STEP 2. Update the history of cutting *L*_*c*_ using the time-series tool tip position ***p***_*d*_, and obtain *L*_*r*_ by volume calculation on the basis of Eq (1).
STEP 3. Generate the differential map *M* from *L*_*r*_ using the endoscopic camera’s location ***p***_*c*_ and orientation ***e***.
STEP 4. Generate the final AE image *C*_*AR*_ by locally shifting the pixel value *C* using *M*.

In the following sections, we describe the details of the methods used in STEPS 2, 3, and 4.

### Differential map between current and target shapes

**Fig 3:**
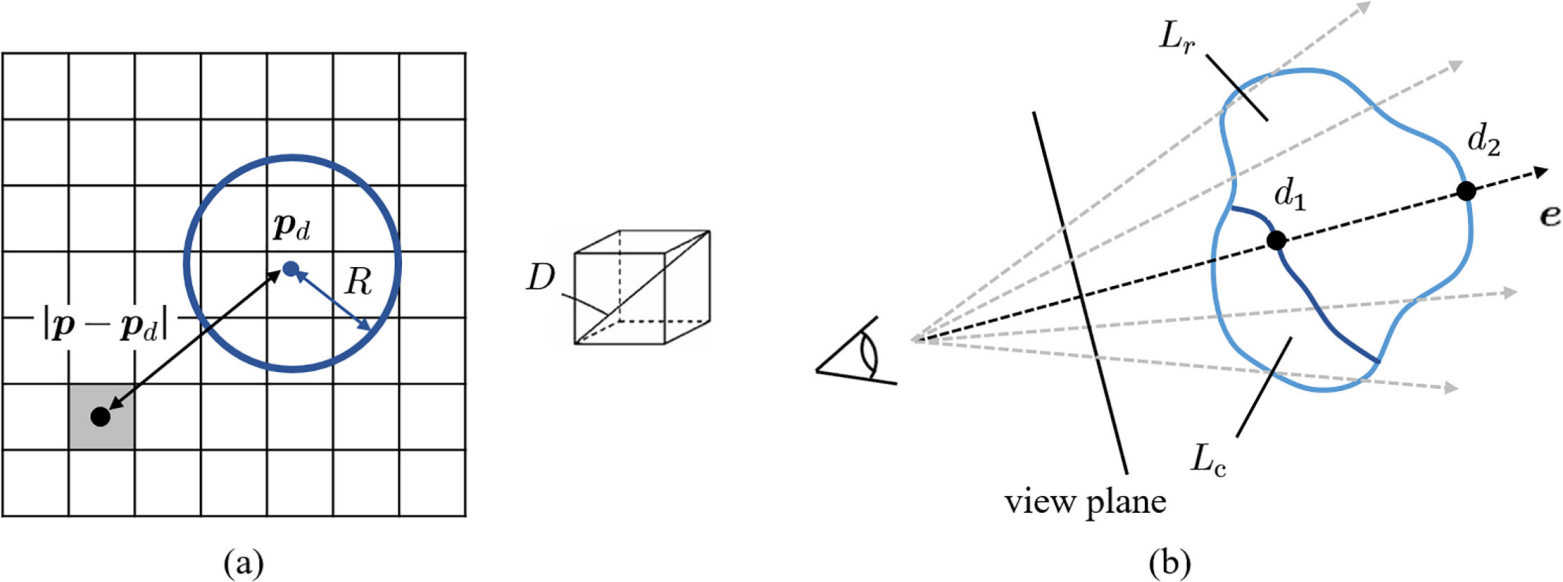
Volume label definition. (a) History of cutting label. The tip of the surgical tool is represented by a sphere, and the current cutting state is stored by the binary voxel values. (b) Definition of remains to be cut in the depth direction. The differential map is used for generating the AR image.

In this section, we explain how the differential map *M* between the current and target shapes is computed. Based on Eq (1), the remains to be cut *L*_*r*_ is defined by volume subtraction of the history of cutting *L*_*c*_ from the region scheduled to be cut *L*_*p*_. *L*_*r*_, *L*_*c*_, and *L*_*p*_ are volumetric binary labels. As mentioned in the previous section, *L*_*p*_ is set by the medical staff during the preoperative planning phase. We first initialize *L*_*c*_ by setting the value *l* = 0 to all voxels where the surgical tool tip has not passed. Here, the tool tip is described by a sphere with a radius *R* to model the tip of a surgical drill, wherein the voxel value *l* in *L*_*c*_ can be updated from the relationship between the central location of the arbitrary voxel and the tool tip position ***p***_*d*_. Fig 3A shows a 2D schematic for this relationship, where ***p*** is the central location of the voxel and *D* is the length of the diagonal line of the voxel. We then define the post-cutting state *l* = 1 based on Eq (2).

$$l=\left\{ \begin{array}{c} \;1\\ \;0 \end{array}\right.\begin{array}{c} \;if\;\delta \geq th\;and\;p_{d}\in L_{p}\\ \;else \end{array}$$ (2)

where *δ* is defined as follows:

$$\delta =\left(R-|p-p_{d}|\right)/D+0.5$$ (3)

This scheme updates the volume label *L*_*r*_ representing the remains to be cut from the time-varying history of cutting *L*_*c*_ during surgery. The proposed differential map *M* to be computed is a 2D depth image of *L*_*r*_ that represents the amount of the remains to be cut in the camera’s line of sight ***e*** spatially registered to the camera image *C*. By applying a simple ray-casting technique [10], we can detect the distance to the deepest point *d*_2_ and the distance to the surface point *d*_1_ in the volumetric space of *L*_*r*_. This process is performed for all pixels of *M*, and the voxels of *L*_*r*_ are scanned in multiple directions by considering the perspective transformation (Fig 3B). The depth value obtained by *d* = *d*_2_ − *d*_1_ is then set as a pixel value of the differential map *M*.

### Overlaying differential map

This section describes the methods for overlaying the differential map on the endoscopic images. During cutting procedures, it is necessary to observe the current partially cut shape of the bone structures. Additionally, soft tissues, vessels, and nerves near the spine should be carefully treated throughout the operation. When generating AE images, we should consider that simple blending of the differential map *M* and the endoscopic image *C* decreases the contrast of both images. Opacity control may make visual confirmation of real endoscopic images more difficult. To preserve the features of the endoscopic image, we do not employ a traditional opacity control, but instead use a color transfer function (*TF*) to locally shift the pixel values for the endoscopic images *C* based on the differential map *M* using Eq (4).

$$C_{AR}(x,y)\leftarrow TF(C(x,y),M(x,y))$$ (4)

In this function, pixel color is evaluated in the hue/saturation/value color space, and the hue *H*(*x*,*y*) for each pixel within the area of *M* is shifted using Eq (5).

$$H(x,y)=\left(H_{max}{-H}_{min})\times M(x,y\right)/M_{max}+H_{min}$$ (5)

where *H*_*max*_ and *H*_*min*_ are the maximum and minimum hue values, respectively, of *C*. *M*_*max*_ is the maximum value for the depth value with regard to *M*(*x*,*y*). As we shift only hue values in this function, visual appearances such as textures and intensities of the endoscopic images can be preserved. For spinal navigation in this study, the allocated hue range was set at *H*_*max*_ = 180 and *H*_*min*_ = 60. This means that the remains to be cut with a certain thickness in depth are shifted to the blue color and that the partially cut area or remains to be cut with a smaller depth value are shifted to the yellow color.

**Fig 4:**
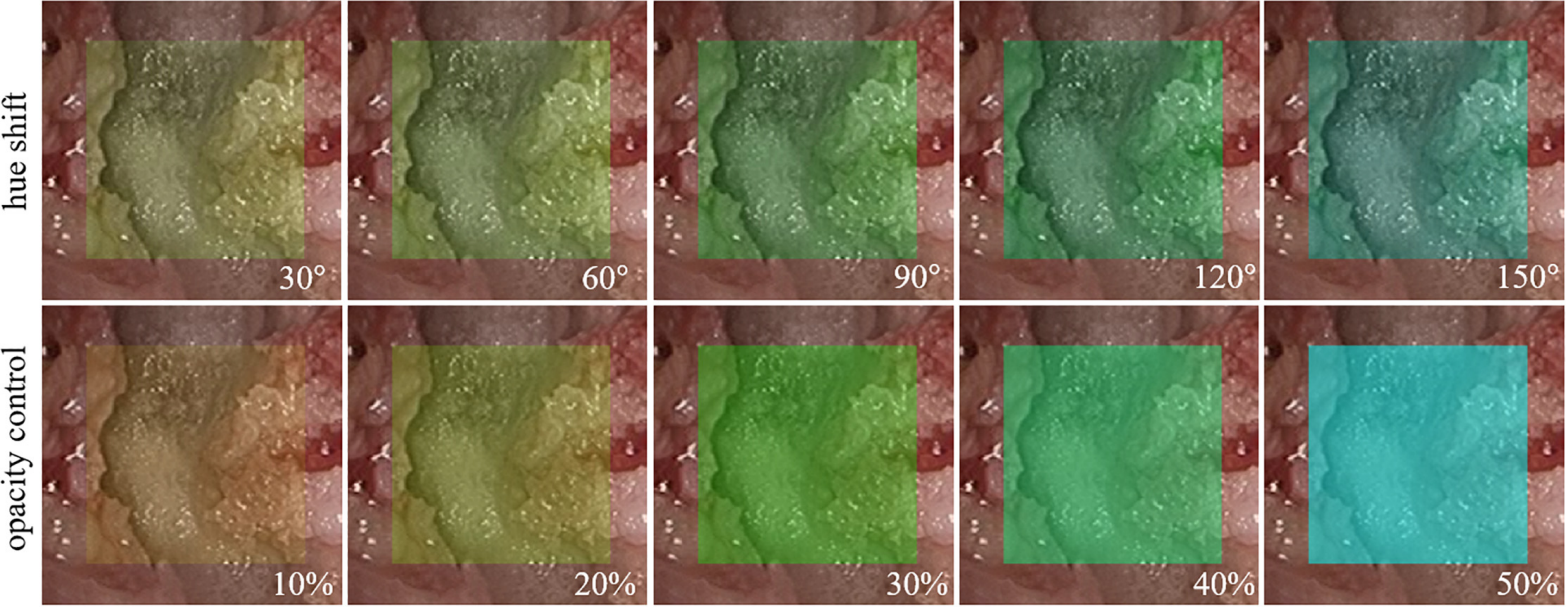
Comparison of color overlay methods. Hue shift in the hue saturation value color space and opacity control applied to the microendoscopic images.

As an example, Fig 4 shows two types of color overlay for microendoscopic images when a rectangular region is used for a cutting target. The upper images are generated by hue shift and the bottom rows are obtained from opacity control with a similar color configuration. In the hue shift model, the contrast of the background is preserved. When the shift value is smaller, the visual appearance more closely resembles that of the original microendoscopic image. Based on the transfer function in Eq (5), the amount of hue shift is controlled by the depth to be cut, which naturally causes surgeons to concentrate on the anatomical structures near the edge of the region scheduled to be cut. In contrast, opacity control fails to preserve the visual appearance of the background while changing the level of the information overlay. The low transparency makes visual confirmation of the real microendoscopic images more difficult.

When the cutting is performed beyond the region scheduled to be cut, additional color overlay can be presented at the incorrect cut regions. During microendoscopic procedures, it is important to inform surgeons of this potential risk as early as possible to avoid drilling in the wrong directions or inducing nerve injury at the deepest point of drilling. To prevent such misoperation, the present study focused on the importance of navigating the remaining depth and correct direction in the incomplete cutting state. The proposed graphical overlay allows for continuous visualization of the remaining area of cutting while providing depth information, which can contribute to precise, evidence-based cutting procedures. Image filters with a simple threshold are also available in the transfer function to preserve specific features such as surgical tools and nerve structures. For example, the original pixel values are used when the pixel values for *C* are close to those of the structures to be filtered. This scheme enables us to achieve information overlay in the generation of the AE images while preserving anatomical features to the greatest extent possible.

### Experimental system design

We implemented a sequence of algorithms using C++, OpenGL, GLSL (Open GL Shader Language), and the software package NVIDIA CUDA (Compute Unified Device Architecture). S1 File includes the C++ source codes for interactive volume cutting, and S1 Movie shows real-time update of spinal CT volume data.

**Fig 5:**
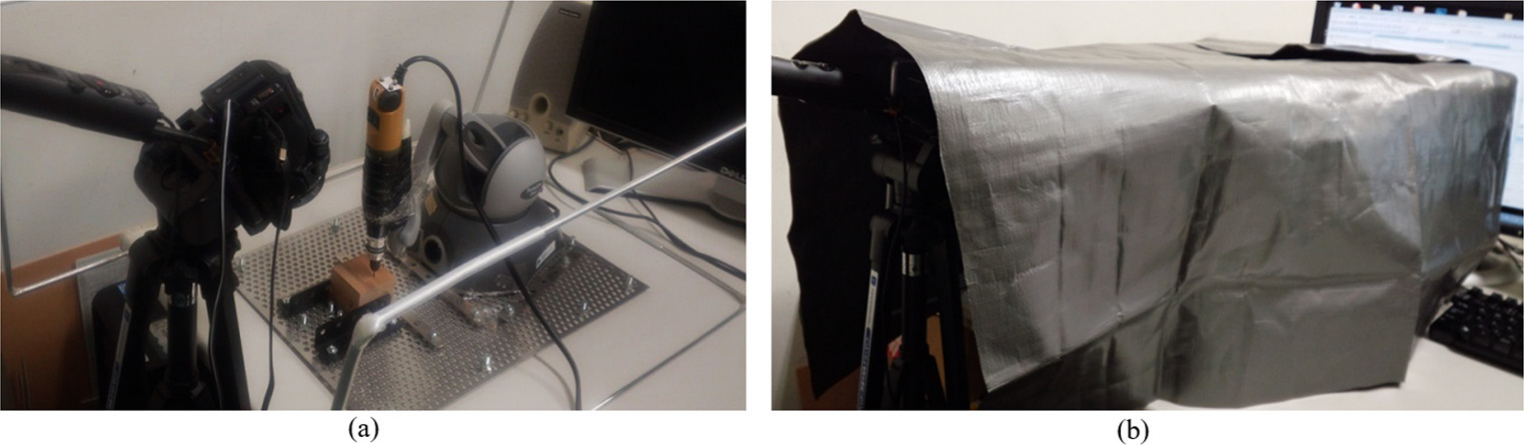
Hardware setup and workspace for experimental system. (a) An electric router mounted on the PHANToM was used for tool tip measurement. (b) The workspace was covered and the subjects could only see camera images displayed in the monitor.

The air drill used clinically for MED is expensive and unfavorable for nonclinical use. We therefore designed an original experimental system using proxy hardware and experimental materials to confirm the potential performance of the proposed AR cutting aid. Here, we substituted the air drill that was normally used during the surgery with an electric router (Proxxon Inc.). This electric router is used for actual cutting training in clinical education. The router position was measured by mounting a PHANToM Omni stylus pen on the electric router. We then obtained the position of the router tip using the orientation/position acquisition function using an OpenHaptics library. In this study, we prepared both wooden blocks and a 3D printer model of the spine, both of which are also used in actual cutting training. Fig 5A shows how we fixed the PHANToM and a wooden block (each side being 50 mm in length) onto a working area. Initial calibration is conducted using a simple calibration tool, which is also a wooden block of the same length containing a 25 mm thin hole in the body. The calibration tool is fixed at the same position on the working area, and the tip position and orientation of the electric router is measured by inserting the tip of the router in the hole. Additionally, the freedom of rotation of the tool tip is restricted because the surgery is performed within metal tubes with a 16-mm diameter. This was an attempt to replicate the limited space and angles for tool operation in real microendoscopic surgery.

Next, images of the object to be cut were captured using a generic video camera (Sony HDR-CX720Vz) as a substitute for an endoscopic camera. Only the portion of the object to be cut could be enlarged and viewed using an endoscopic camera during actual surgery. The surgical field is normally illuminated using a light source positioned on the tip of the endoscopic camera. A metal frame was created to generate the same light and space conditions of the surgical field. Based on this observation, we constructed an experimental environment wherein the object scheduled for cutting is not directly visible from the outside because of the electric router and by wrapping objects for cutting in vinyl sheets, as shown in Fig 5B. Additionally, a visual field of a microendoscopic surgery was replicated by masking all areas outside the encircled area of the camera images. A light source was then fashioned by mounting a light on the side of the video camera. Lens distortion was small and of negligible consequence in this experimental system.

## Results

### Overlay visualization results

**Fig 6:**
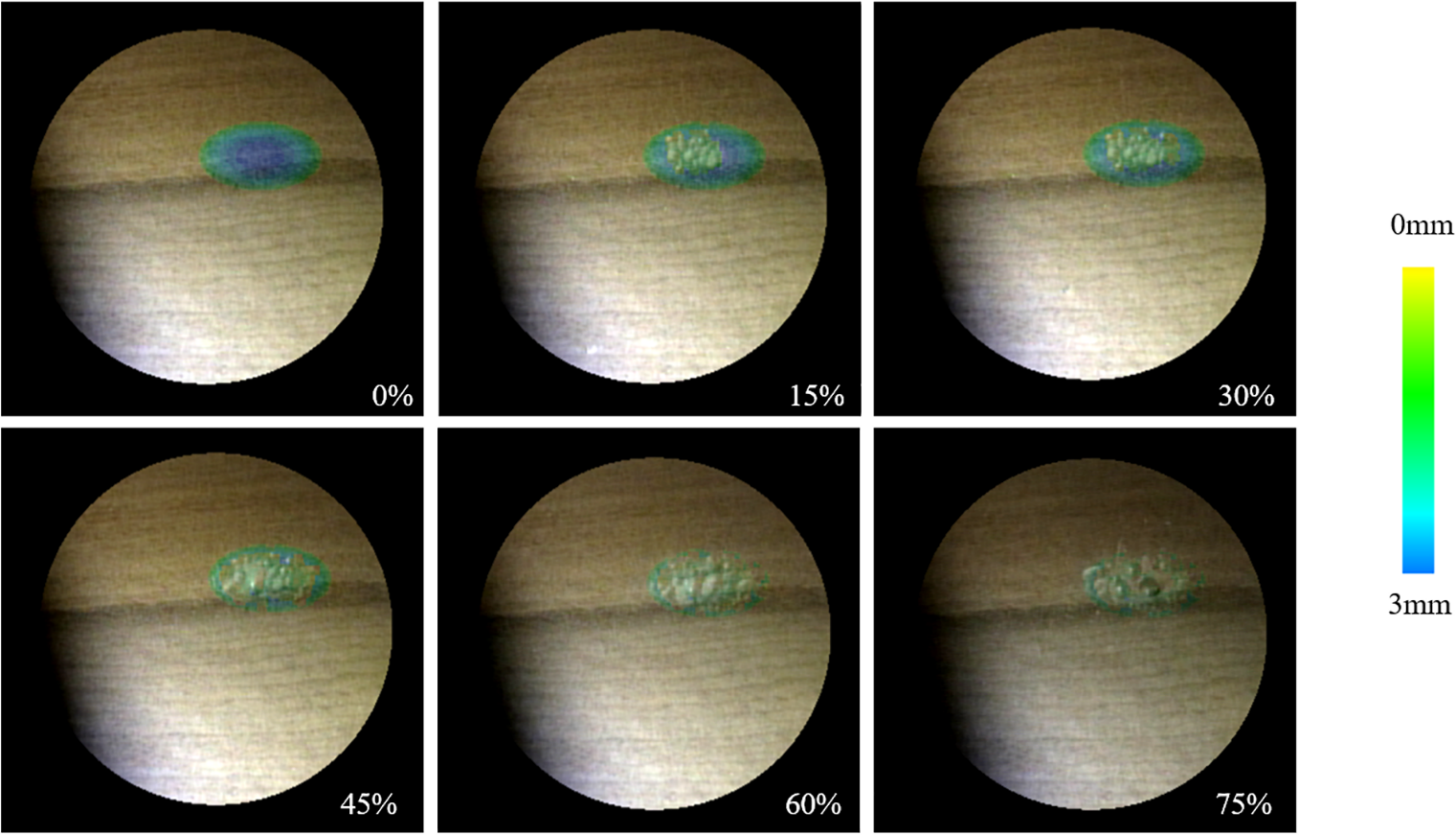
Augmented camera images as the cutting operation progresses. The color represents the amount of cutting remaining for the target shape.

We first confirmed the efficacy of the AE image overlaying differential map *M* during the cutting operation using the designed experimental system. In this test, a cutting operation was performed using an electric router on a 50-mm cuboid wooden block up to 15%, 30%, 45%, 60%, and 75% for the target shape. The AE images obtained are shown in Fig 6, and the time-varying color shift is demonstrated in S2 Movie. The intensity and saturation of the camera images were preserved, and a heat map on the right side of Fig 6 was used to render the range of hues to reflect *H*_*max*_ = 240 and *H*_*min*_ = 60. In this case, the amount of remains to be cut for the blue area was 2.75 to 3.00 mm, and the amount of remains to be cut for the yellow area was 0.00 to 0.25 mm. If the entire region scheduled to be cut was actually cut, the color shift was not applied. The results show that the time-varying color shift in the camera images as the cutting operation progresses is thought to be a useful aid for cutting.

**Fig 7:**
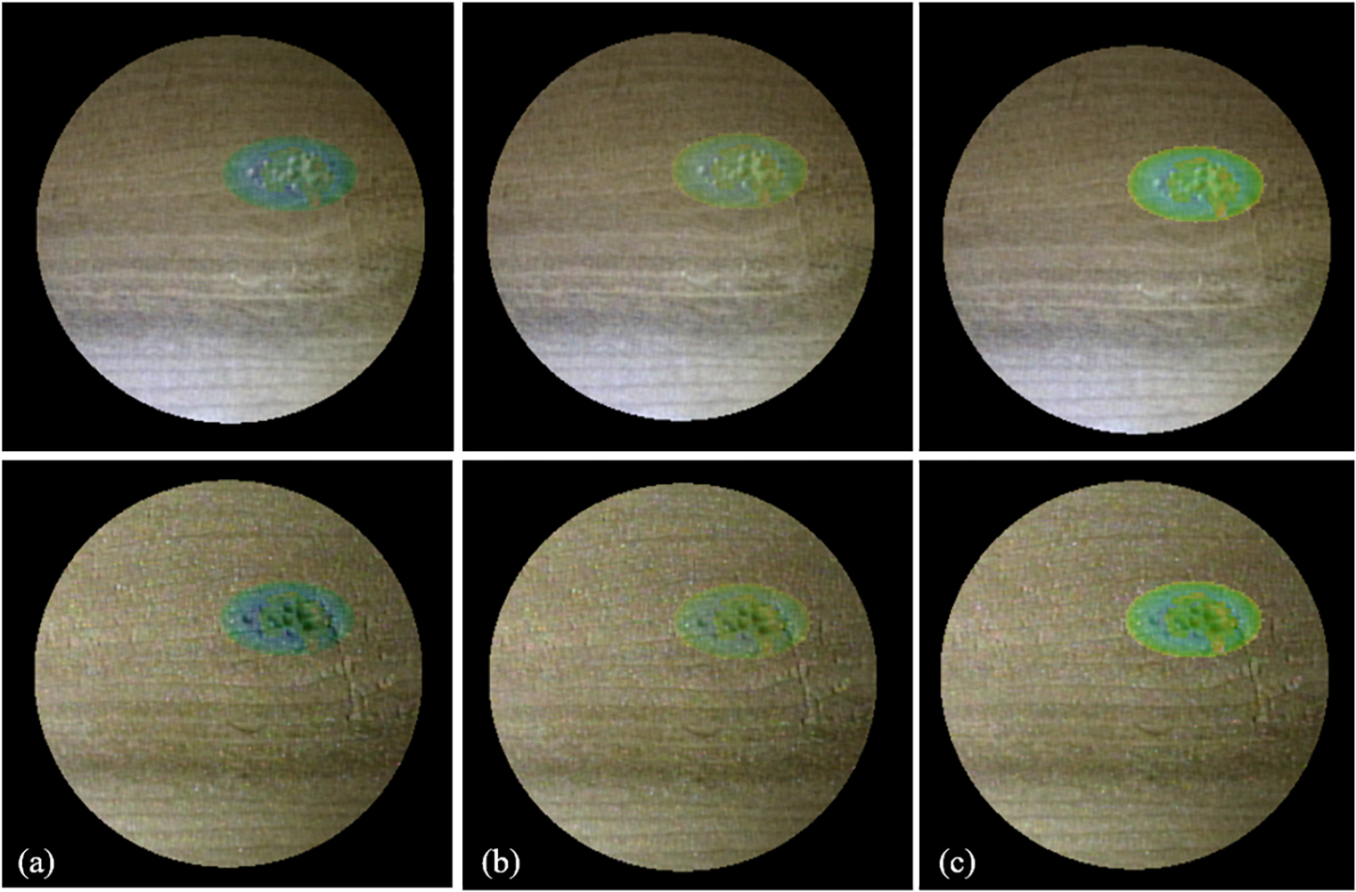
Augmented images for cutting aid. (a) Local color shift and alpha blending with (b) 10% and (b) 20% opacity values. The local color shift achieves better visibility of textures and shapes in different illumination conditions.

Next, we confirmed the efficacy of the information overlay wherein the pixel values were locally shifted by the proposed framework. For this test, we cut a small amount of a wooden block with the same shape on top. The results with the color shift and blending operation were compared under the different illumination conditions. Fig 7A shows the results of locally shifting the pixel values *C*(*x*,*y*) using the proposed technique. For comparison, Fig 7B and 7C show the results obtained using conventional techniques to simply blend *C* and *M* using the same color mapping. *M* was made to become semi-transparent and then blended with *C*. The opacity was set at 10% in Fig 7B and 20% in Fig 7C. We also prepared two examples: (1) the illumination conditions, including the direction of light illumination and the direction of the camera’s line of sight, were held constant at 0° (upper images of Fig 7) and (2) at +30° (bottom images of Fig 7). The locations of the lights on the left and right sides of the camera were changed, and the wooden blocks were set at the center.

In Fig 7A, the wooden grains are visible, and the shading and texture of the wooden surface where the cutting was to be performed are clearly visible. In contrast, poor visibility of the textures and shapes of the objects for cutting was found when using the previous technique. Overlaid heat maps used as cutting aids were also difficult to confirm visually with the previous technique when the transparency was increased. In contrast, we confirmed that the color distribution in the proposed method changes in response to the illumination conditions.

**Fig 8:**
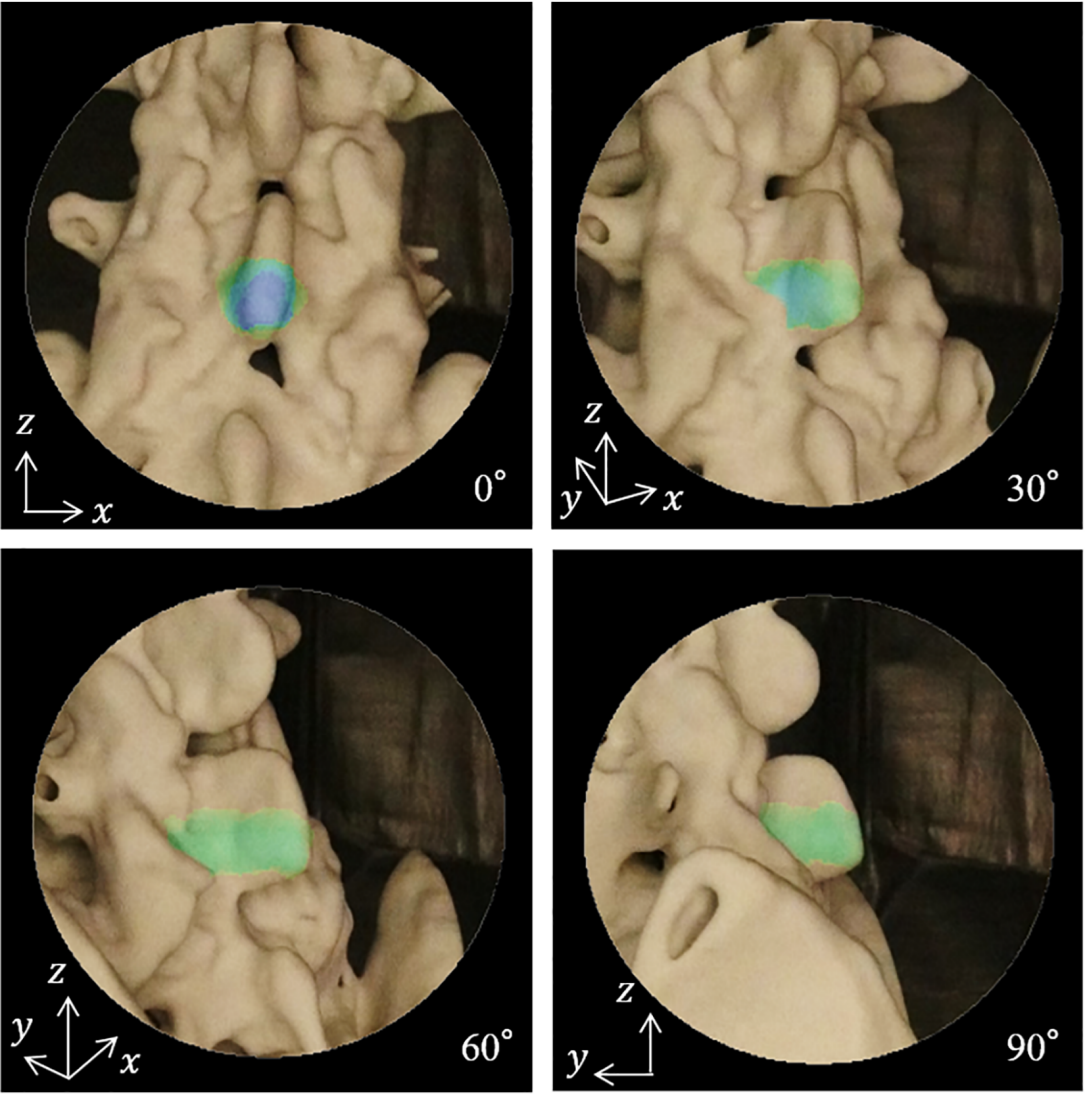
AE images of a 3D printer model made from CT images with different camera orientations. The direction and depth that should be cut on the spinal structure are visualized using the heat map.

We also generated AE images for spinal shapes using patients’ CT images. This use of the medical images were approved by Wakayama Medical University’s Ethics Committee, and the experiments were conducted after anonymization of the patient information. We performed overlay visualization on the region scheduled to be cut using a plaster-type 3D printer model created from the preoperative CT images. Additionally, we replicated the region that was cut during surgery for *L*_*p*_ based on preoperative and postoperative CT volume data. *L*_*p*_ (= *L*_*r*_) was generated for the camera images, whereas the orientation of the camera was changed from 0° to 90° in 30° increments from the center for the model. Fig 8 shows the AE images obtained. There were many regions where the overlaid color was yellow-green, highlighting the regions scheduled to be cut when observed from 90°. This shows that the amount of cutting in the *x*-direction should be reduced. We also observed that the blue regions increased, which signifies that the long cylindrical region should be scheduled to be cut in the *y*-direction while the direction is moving from 90° to 0°. We were able to replicate changes in the appearance of the region scheduled to be cut. The direction and depth that should be cut could be designated via generation of *M*, which reflects the camera’s orientation during endoscopic surgery where only a localized view can be obtained. When the direction in which the cutting should progress differs from the orientation of the endoscopic camera, the surgeon would thus be able to more easily detect misalignment and better understand what areas need to be cut, in which direction, and to what degree.

### Cutting experiments

The next experiment aimed to verify that the proposed AE images are effective as a cutting aid when performing the cutting on wooden blocks of predetermined shapes. Microendoscopic procedures require high levels of technical skill and anatomical knowledge that depend on the individual surgeon’s skills. This was the primary motivation of the present study, and the AR cutting aid was designed as an objective support tool. In this context, we considered that the performance of the developed techniques should be first evaluated independent from the current surgeons’ skills, and nine non-experts in their 20s or 30s (all right-handed) were chosen. The experiment was then conducted in a non-clinical setting to confirm the performance of the cutting aid in basic cutting procedures with an electric router.

**Fig 9:**
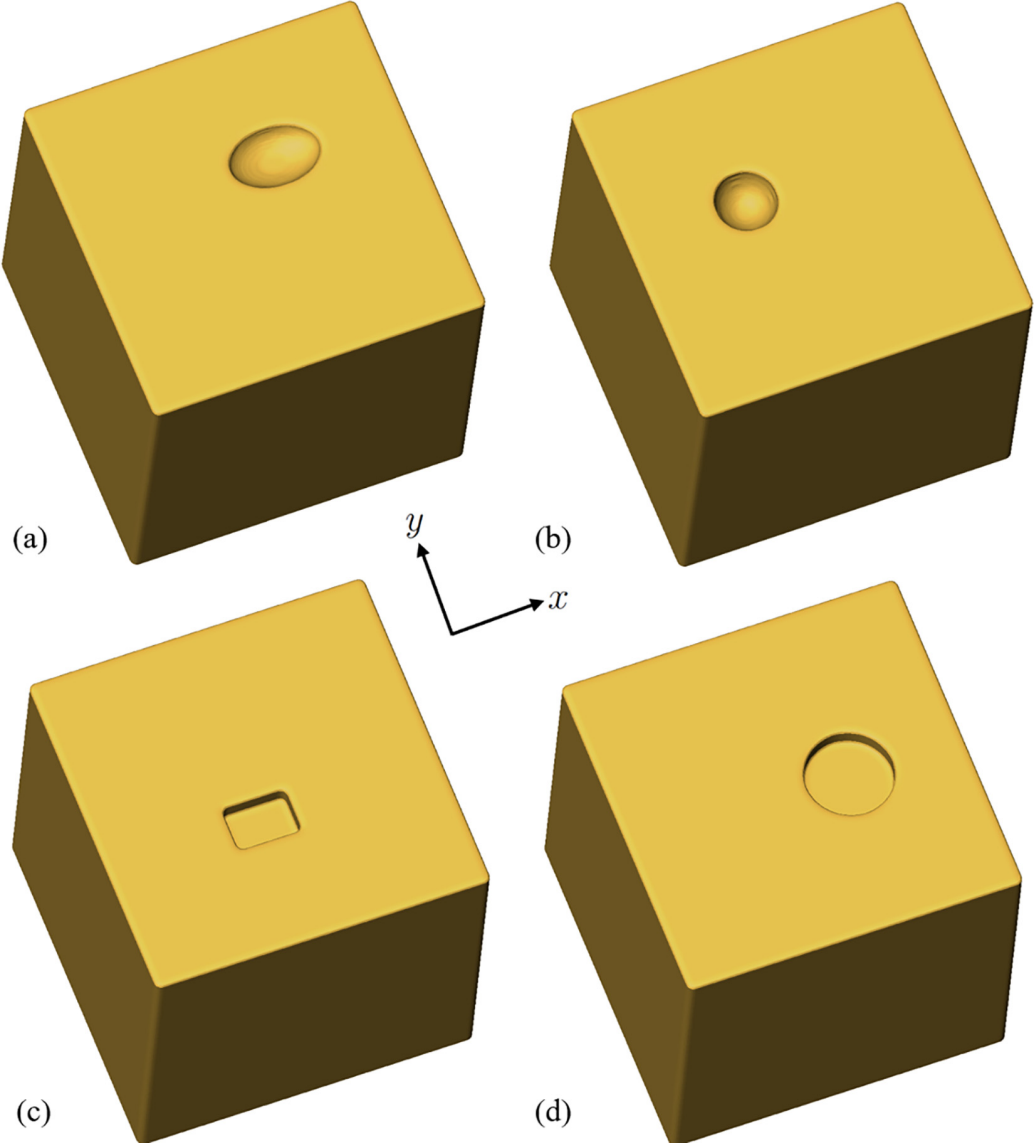
VR images of four target shapes for cutting experiments. (a) Semi-ellipsoid, (b) hemi-spherical, (c) cuboid, and (d) columnar.

During the trial, the participants underwent cutting operations using two methods to achieve the target shape. The first method applied the existing technique wherein the video camera images and the VR images were lined up and shown side by side. The second method involved the proposed technique, wherein the pixel values of the video camera images are locally altered. In both the existing and proposed techniques, the objects to be cut were visualized by changing the color of the region scheduled to be cut. In this experiment, four target cutting shapes were prepared as shown in Fig 9. The regions scheduled for cutting were semi-ellipsoid (12,919 voxels, 202.0 mm^3^), hemi-spherical (12,707 voxels, 198.5 mm^3^), rectangular (12,312 voxels, 192.4 mm^3^), and columnar (24,348 voxels, 380.4 mm^3^). Each VR image was also presented as an existing cutting aid with VR images.

To avoid bias from the order of effects, the experimenter presented two types of cutting aids in a random order for each of the participants and in the four trials. In the experiment, the participant sat in a chair facing the main monitor. An auxiliary monitor for presenting the side-by-side virtual image was placed on the left side neighboring the main monitor. The wooden block was then placed between the main monitor and the participant. The height of the chair was adjusted to ensure natural placement of the hand at the same level of the wooden block. The experimenter also instructed the participants not to look at their hands when cutting and to cut while observing only the displayed images—in the same way they would if performing endoscopic surgery. Additionally, a red square mark was displayed at the top of the video camera for 10 sec per minute, during which time the participants were instructed to stop cutting. We expected this setup to bring about encouraging effects, such as the participants being able to focus their attention naturally on the display to simulate bleeding in the same manner as would happen during surgery. After the cutting operation was completed, we recorded the cutting history label *L*_*p*_; that is, the regions that were cut. The experimenter also measured the location of the electric router tip within the experimental system while the cutting operation was being performed.

We computed three evaluation indices from the obtained data and performed an evaluation while comparing the proposed and existing techniques. The required cutting time *E*_*t*_ represents the cutting time needed from start to finish. The cutting was completed when the participants themselves determined that the overlaid color had disappeared from the region scheduled to be cut. The total moving distance of the electric router tip *E*_*d*_ (the total movement distance for the tip) represents the index provided in Eq (6), which uses the location of the electric router tip ***p***_*d*_ (in millimeters).

$$E_{d}=\sum_{t}\left|p_{d}(t)-p_{d}(t-1)\right|$$ (6)

If this value is small, it is thought that the participants were able to cut efficiently and were sure of which location should be cut. We also considered the similarity between the region that was already cut and the region scheduled to be cut using Eq (7).

$$E_{s}=2|L_{p}\wedge L_{c}|/\left(\left|L_{p}|+|L_{c}\right|\right)$$ (7)

The value of *E*_*s*_ ranges from 0 to 1 and evaluates the similarity between *L*_*c*_ and *L*_*p*_ in terms of volume and location. If the value is close to 1, it can be considered that the cutting operation could be performed near the target with a small shape error. The experimental protocols and analysis of the results are summarized as follows.

- Align the positions from the differential map and the camera images using the four peak points on the top of the wood as reference points.
- Have the research participants become familiar with the feeling associated with cutting the wooden blocks by freely cutting the blocks until they are sufficiently familiar with operating the electric router.
- Position the wooden block used to calibrate the axes and the default position on the workstation with the PHANToM.
- Calibrate the axes and the default position for the electric router.
- Start cutting after fixing the wooden blocks with tape so they cannot move when switching out the wooden block used in the S calibration with the wooden block to be cut.
- Mark the end of the cutting operation as the point at which the research participants determine that cutting all of the regions scheduled has been completed. Record the time required for cutting *Et*.
- Calculate the total movement distance *E*_*d*_ from the router tip.
- Calculate the similarity of *E*_*s*_ between the obtained cutting label and the regions scheduled to be cut.

**Fig 10:**
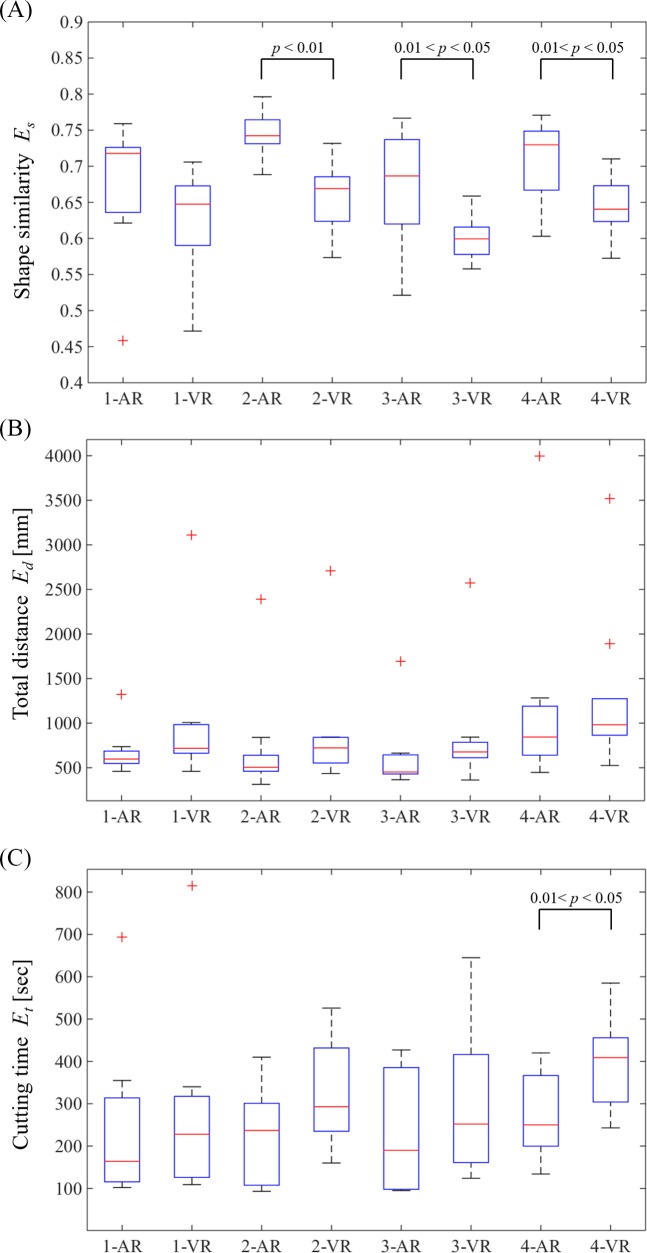
Evaluation results of cutting aid using the proposed AR images and VR images for comparison. (a) Cutting time *E*_*t*_, (b) total distance of the electric router tip *E*_*d*_, and (c) shape similarity of the target region *E*_*s*_.

Fig 10A shows the cutting time *E*_*t*_ required, and Fig 10B shows the total movement distance *E*_*d*_ for the electric router tip. Fig 10C shows the similarity measure *E*_*s*_ for final *L*_*c*_ and *L*_*p*_. The box plots include the minimum, first quartile (Q_1_), median (Q_2_), third quartile (Q_3_), and maximum. The minimum and maximum scores are represented after outliers have been rejected. Values larger than (Q_3_ − Q_1_) × 1.5 + Q_3_ or smaller than Q_1_ − (Q_3_ − Q_1_) × 1.5 were regarded as outliers. The plotted whisker extends to the minimum (or maximum) value that is not an outlier. The cross indicates an outlier that is out of 99.3% coverage if the data are normally distributed.

Fig 10A shows the trends of having shorter required cutting times when using the proposed technique. There was a significant difference in region 4 by one-way analysis of variance (5% significance level). As shown in Fig 10B, the average total movement distance for the electric router tip was shorter when the proposed technique was used. However, there was no significant difference between the two techniques. Fig 10C shows that interestingly, the similarity between the cutting regions and the regions scheduled to be cut was greatest in regions 2, 3, and 4 when the proposed technique was used; significant differences were found in the three cases. In contrast, although no characteristic differences were found between the two techniques used in region 1, the average values showed greater similarity in each of the four regions when the proposed technique was used.

### Implementation of software in the clinical navigation system

**Fig 11:**
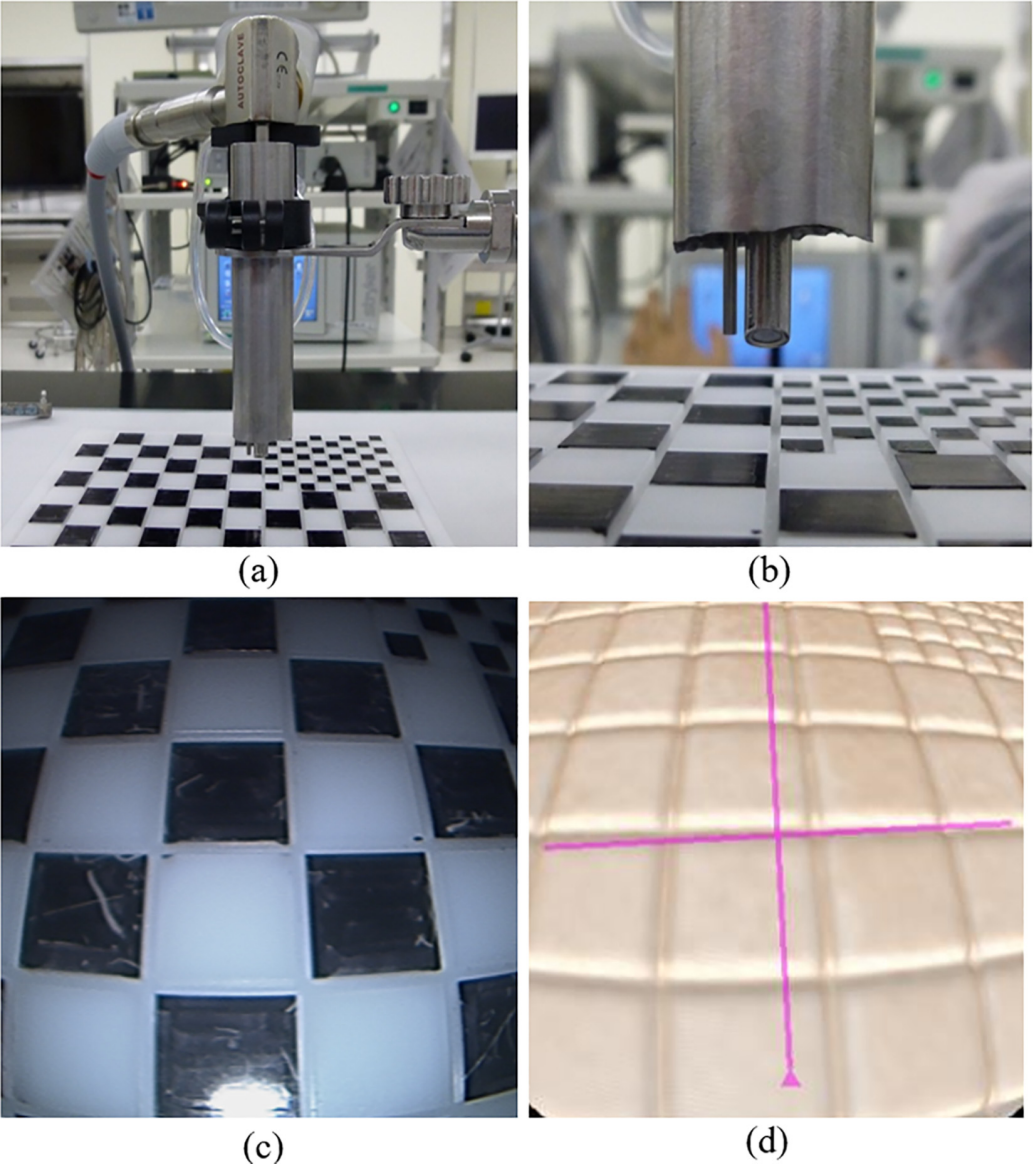
Virtual image generation using StealthStation. (a) Metal tube with 16-mm diameter, (b) microendoscope attached to metal tube, (c) microendoscopic image, and (d) calibrated virtual image reflecting microscopic lens properties.

To confirm the feasibility of the proposed AR guidance techniques, the developed software was integrated into an intraoperative navigation system (StealthStation S7; Medtronic, Minneapolis, MN) that is clinically used at Wakayama University. In this system, the endoscope position ***p***_*c*_, endoscope orientation ***e***, and drill tip position ***p***_*d*_ are obtained from the optical tracking subsystem equipped with the Polaris. Fig 11 shows the physical setup of the hardware, the microendoscopic image, and the virtual image reflecting the microscopic lens property. Four reflective markers were attached to the end of the microendoscopic camera and the reference probe. The tip of the probe can be uniquely computed from the position of the reflective markers. Notably, these tools and marker settings are based on the standard configuration provided by StealthStation and clinically used for intraoperative navigation. The camera parameters measured at 100 Hz are used for rendering virtual images. The endoscopic camera images contain distortion generated from optical characteristics of the camera. To reflect the lens characteristics of the microendoscope, a previously-developed panoramic transformation [27] was performed in the volume rendering scheme, and the graphical overlay was generated with distortion to match the live target (Fig 11D).

**Fig 12:**
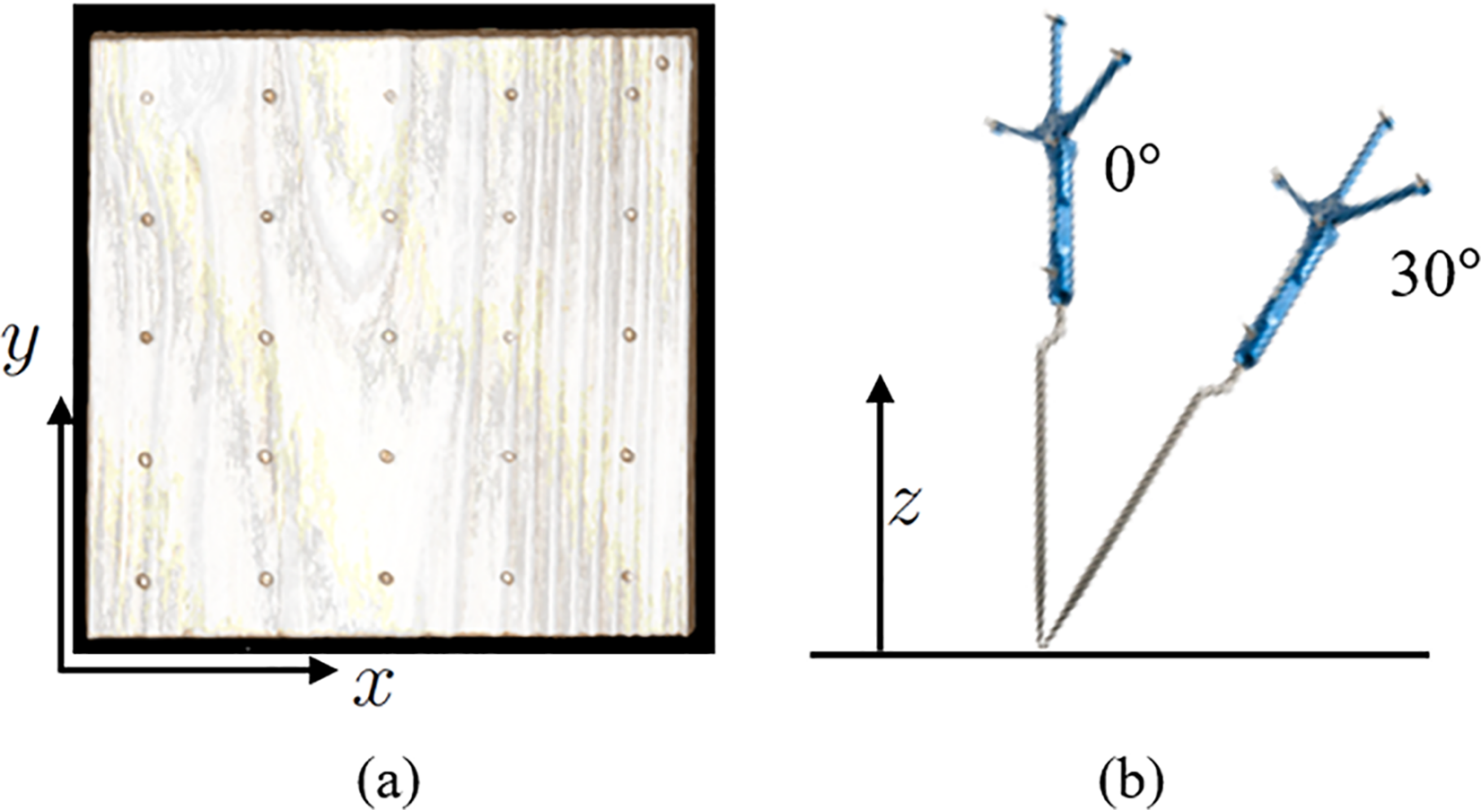
Registration accuracy evaluation using clinical navigation system. (a) Evaluation points marked on the top of a wooden block and (b) reference probe attached with reflective markers.

The calibration accuracy of the probe’s tip position provided by StealthStation S7 was examined. In the experiment, a 120-mm cuboid wooden block (Fig 12A) was prepared and installed on a workbench in an operating room. 5 × 5 small hollows were created at 1 cm intervals on the surface as evaluation points. The initial calibration was conducted by measuring the four corner points (P_1_,…,P_4_) on the top of the block. The distance between the block and the optical tracking sensor of StealthStation was set at 1.8 m. The experimenter pointed at the 5 × 5 evaluation points with the test probe, and the tip position was measured in 5 s for each point while keeping the probe static and perpendicular to the top surface of the wooden block as shown in Fig 12B. Next, the experimenter fixed the probe at an angle of 30° to the surface, which is a possible orientation of the surgical drill during microendoscopic surgery. The distance between the block and the optical tracking sensor position was changed to 2.0 m, and the same measurement protocol was conducted with the two orientations of the test probe.

The positional errors on the evaluation points were computed to evaluate the calibration accuracy. The center of the four registration points (P_1_,…,P_4_) was used as the fiducial position, and the positional errors were calculated as the absolute distance between the average of the measured data for each point and the corresponding ideal position uniquely defined from P_1_,…,P_4_. Table 1 summarizes the median and standard deviation of the positional errors of the *x*, *y*, and *z* directions in the calibrated world coordinates and the Euclidian distance. The results show that stable acquisition of the tip position with an average 0.52 mm positional error was possible. Because the voxel size of the CT images is 0.5 mm, this result demonstrates that the history of cutting *L*_*c*_ can be updated with approximately one voxel spatial error using the existing machine with the clinically used optical marker settings.

**Table 1:** Positional errors on tip of reference probe.

		*d* [mm]	*x* [mm]	*y* [mm]	*z* [mm]
**1.8 m**	**0°**	0.39 ± 0.18	0.24 ± 0.18	0.12 ± 0.14	0.23 ± 0.12
	**30°**	0.55 ± 0.36	0.33 ± 0.27	0.26 ± 0.20	0.23 ± 0.25
**2.0 m**	**0°**	0.52 ± 0.23	0.21 ± 0.20	0.32 ± 0.23	0.18 ± 0.14
	**30°**	0.55 ± 0.30	0.38 ± 0.26	0.32 ± 0.24	0.18 ± 0.15

## Discussion

In this paper, the concept of an AE image that overlays shape changes in microendoscopic cutting procedures was proposed. The proposed framework overlays differential maps between the current and target shapes in the endoscopic images, and the remains of cutting are visualized on the endoscopic image. Our findings imply that the depth and direction of the scheduled cut can be provided as augmented microendoscopic images, with which the surgeons are able to more easily detect misalignment and better understand what areas need to be cut. The cutting experiment was conducted with the help of participants, and the performance of cutting aids was compared by the proposed AE images and the existing VR images. We were able to confirm the efficacy of the proposed technique from the standpoints of similarity between the cutting regions and the region scheduled to be cut, the total movement distance for the electric router tip, and the time needed to accomplish the cutting.

Although little differences were found in region 1, the proposed technique showed favorable results based on the average values with regard to all of the indices. One reason why no differences were found in region 1 could be the semi-ellipsoid shape of this region, which was defined as a complex shape. It is also possible, however, that it may have been due to the large differences in skill levels among the research participants; the cutting operation performance varied greatly based on the individuals’ skill levels. Although the outlier was particularly large for movement distances of the electric router in each of the regions, this was because one of the research participants frequently moved the electric router a small distance while cutting, thereby skewing the results.

In this cutting experiment, the median values of the shape similarity were improved by 7%, 8%, 9%, and 8%, respectively. Regarding absolute size, in the case of region 3 with a 48 × 32 × 8 voxel rectangular shape, for instance, 8% corresponds to an approximate deviation of 4 voxels in the *x* direction. Although this improvement is relatively moderate compared with the side-by-side virtual image presentation, we consider that this result is due to the fact that simple geometries were used as cutting targets to reduce bias from individual cutting skills. In microendoscopic surgery, spinal bones and cutting targets have more complex shapes. Actually, even a small cutting error on the surface of spinal structures may result in misdrilling and disorientation in approaching the deepest point to be treated. Because nerve structures are in contact with the cutting target of the spine and because the visible area during microendoscopic procedure is severely limited, reducing the cutting error by even 1 mm can contribute to a reduced risk of surgical incidents.

The last experiments involving clinical implementation of the software showed the feasibility of the proposed AR-assisted cutting framework in the clinical setting. Endoscopic camera distortion was reproduced to match the live target with the virtual images. Further experiments are required to confirm the clinical validation of the developed system. The reproducibility of the virtual images and their registration to the real microendoscopic images should be examined quantitatively. The clinical benefit of the AE image over standard procedures is an important topic to be investigated. AR guidance has the potential benefit of serving as a more objective intraoperative support tool and procedural training tool in clinical education. We plan to perform a clinical study involving use of the developed system as an intraoperative support tool during microendoscopic surgery.

## Supporting Information

S1 File C++ source codes for interactive volume cutting. (ZIP)

S1 Movie Real-time update of spinal CT volume data (MPG)

S2 Movie Augmented endoscopic images in the cutting experiments (MPG)
