# Supplementary figures and images for: Augmented Endoscopic Images Overlaying Shape Changes in Bone Cutting Procedures
Source: PLoS One. 2016 Sep 1;11(9):e0161815. doi: 10.1371/journal.pone.0161815 (PMC5008631; doi:10.1371/journal.pone.0161815)

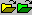

Supplement: S1 File — (ZIP) [file pone.0161815.s001.zip › S1 File/Source/res/Toolbar.bmp]
